# One-Pot Orthogonal Dual Functionalization of mi3 Self-Assembling Protein Nanoparticles via Sortase A and SpyCatcher/SpyTag Ligation

**DOI:** 10.1021/acs.bioconjchem.6c00173

**Published:** 2026-06-25

**Authors:** Liqiang Wei, Hongfei Wang, Chunyue Du, Antony Kam, Shining Loo

**Affiliations:** 1 Wisdom Lake Academy of Pharmacy, 122238 Xi’an Jiaotong-Liverpool University, Wuzhong No. 111, Renai Road, Suzhou, Jiangsu 215123, People’s Republic of China; 2 Department of Biosciences and bioinformatics, School of Science, 122238 Xi’an Jiaotong-Liverpool University, Wuzhong No. 111, Renai Road, Suzhou, Jiangsu 215123, People’s Republic of China

## Abstract

We present a one-pot approach for orthogonal dual functionalization of mi3 self-assembling protein nanoparticles by combining SpyCatcher003/SpyTag003 isopeptide bond formation with Sortase A-mediated transpeptidation. The nanoparticles were engineered with N-terminal SpyCatcher003 domains and C-terminal LPETGGH motifs, enabling site-specific conjugation of distinct cargo molecules. To overcome expression limitations, an N-terminal vesicle nucleating peptide (VNP6) was incorporated, which enhanced protein yields while maintaining nanocage integrity. Critical to this strategy, the calcium-independent Sortase A variant (SRT7+) was employed to avoid calcium-induced nanoparticle aggregation, enabling orthogonal ligation under physiological conditions. The platform enabled simultaneous conjugation of HER2-targeting affibodies and fluorescent probes in a single reaction, and these dual-functionalized self-assembling protein nanoparticles exhibited specific cellular uptake in HER2-positive cancer cells. This integrated one-pot approach proved scalable and modular for engineering protein-based nanomaterials for targeted therapy and diagnostics.

## Graphical abstract

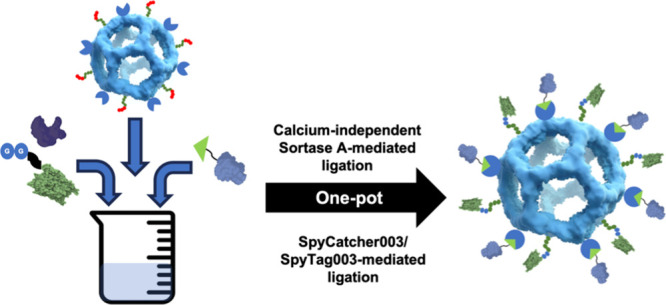

## Introduction

Self-assembling protein nanoparticles, including ferritins, virus-like particles, encapsulins, and computationally designed protein structures, have emerged as versatile platforms for bionanomaterials due to their intrinsic homogeneity, biocompatibility, and abundance of modifiable interaction sites.^1,2^ These assemblies, typically composed of multiple copies of one or few protein subunits, self-organize into highly ordered architectures with distinct internal and external surfaces.^1,2^ Their precise, well-defined geometry renders them particularly useful as scaffolds for displaying or encapsulating cargo, thereby enabling applications in drug and gene delivery, enzymatic catalysis, antigen presentation, and assembly of higher-order functional complexes.^1–6^

A central challenge in exploiting self-assembling protein nanoparticles lies in achieving multisite functionalization with defined stoichiometry and spatial distribution. Conventional single-reaction bioconjugation strategies often suffer from competitive interactions at multiple modification sites, resulting in heterogeneous assemblies with variable cargo distribution and substoichiometric loading.^2,7^ Genetic fusion approaches offer direct incorporation of functional domains but frequently disrupt self-assembly, compromising cage formation or structural stability.^2,7^ Consequently, post-assembly functionalization has become the preferred strategy, preserving nanoparticle integrity while enabling precise cargo attachment.^7^ However, current approaches remain limited. Single-reaction strategies achieve only monofunctionalization, while sequential conjugation requires multiple purification cycles, reducing scalability and material recovery. To overcome these limitations, recent research has increasingly explored orthogonal bioconjugation systems that proceed simultaneously without mutual interference, enabling controlled, multicargo functionalization in a single-reaction vessel.^8^

The computationally designed dodecahedral self-assembling protein nanoparticle mi3 exemplifies an ideal platform for orthogonal dual functionalization.^8–10^ mi3 exhibits exceptional thermodynamic stability and possesses strategically positioned N- and C-terminal regions accessible for modification.^8,11^ Studies revealed that these terminal domains are optimally spaced and oriented to accommodate orthogonal modifications without disrupting assembly.^8^

A major advancement in spontaneous bioconjugation technology is the SpyCatcher003/SpyTag003 (SC3/ST3) system.^12–14^ This third-generation system, derived from the CnaB2 domain of streptococcal fibronectin-binding protein, comprises a 13-amino acid peptide tag and a 116-amino acid protein domain that spontaneously form an irreversible isopeptide bond.^12,13,15^ The reaction proceeds through nucleophilic attack of the lysine residue in SpyCatcher003 (K31) on the carboxyl group of aspartate in SpyTag003 (D7), creating a covalent cross-link that is resistant to thermal, proteolytic, and chemical denaturation.^12^ Critically, this system achieves >90% coupling efficiency within minutes under physiological conditions (neutral pH, 37 °C) without requiring catalysts, cofactors, or external energy input.^12^ The rapid kinetics, high selectivity, and catalyst-free nature of SC3/ST3 make it an attractive platform for protein nanoparticle functionalization.^10,16,17^

While mi3 dual functionalization has been achieved with split-domain SpyCatcher/SpyTag and DogCatcher/DogTag platforms,^8^ a key limitation of such protein-based "catcher" systems is the substantial steric burden they impose on the nanoparticle surface. For instance, the DogTag (14-amino acid peptide) and its ∼15 kDa DogCatcher binding partner collectively create a molecular "footprint" that can restrict functionalization density, compromise nanoparticle solubility, and alter other crucial physical properties, particularly for high-valency or multidomain functionalization. To address these limitations and expand the toolkit for precision engineering multifunctional protein nanoparticles, the development of alternative orthogonal systems is thus highly desirable.

Sortase A, a calcium-dependent cysteine transpeptidase from *Staphylococcus aureus*, and its calcium-independent variants (such as SRT7+) provide a mechanistically complementary approach to SC3/ST3.^18–23^ Sortase A recognizes substrates containing the pentapeptide sorting motif LPXTG (where X is any amino acid) and catalyzes transpeptidation through a two-step mechanism: (1) nucleophilic attack by a catalytic cysteine on the peptide bond between threonine and glycine, forming a thioester intermediate, and (2) nucleophilic attack by an oligoglycine-containing substrate, displacing the enzyme and forming a new peptide bond.^20,21,24^ This mechanism enables site-specific, orientation-controlled conjugation of diverse cargo, including fluorescent dyes, small-molecule drugs, targeting peptides, and imaging agents.^20,21,24^

Here, we describe the integration of SC3/ST3 and calcium-independent Sortase A to achieve one-pot dual functionalization of mi3 self-assembling protein nanoparticles. We demonstrate successful simultaneous conjugation of HER2-targeting affibodies and fluorescent probes, with validation of specific cellular uptake in HER2-positive cancer cells. This integrated platform provides a scalable, modular approach for engineering multifunctionalized self-assembling protein nanoparticles for biomedical applications.

## Results

### Design and Engineering of Dual-Functionalized mi3 Self-Assembling Protein Nanoparticles

Previous studies have demonstrated that mi3 self-assembling protein nanoparticles possess surface-exposed N- and C-termini, making them ideal scaffolds for dual functionalization.^8^ To create a versatile platform capable of orthogonally recruiting two distinct cargos via independent ligation mechanisms, we engineered each mi3 subunit to incorporate two complementary bioconjugation strategies: the SpyCatcher003/SpyTag003 (SC3/ST3) system and Sortase A-mediated ligation.

Specifically, we genetically fused SC3 to the N-terminus of each mi3 subunit while appending a Sortase A recognition motif (LPETGGH) to the C-terminus, yielding the bifunctional construct SC3-mi3-LPETGGH. To minimize steric constraints at functional module interfaces, we incorporated flexible Gly-Ser linkers at each junction. In this design, SC3 enables spontaneous isopeptide bond formation with ST3-modified cargos, whereas Sortase A catalyzes transpeptidation by cleaving the LPETGGH sequence between threonine and glycine residues and subsequently ligating molecules bearing N-terminal diglycine motifs. Figure 1 illustrates the design strategy and conjugation schemes for both sequential and one-pot dual functionalization approaches.

**Figure 1:**
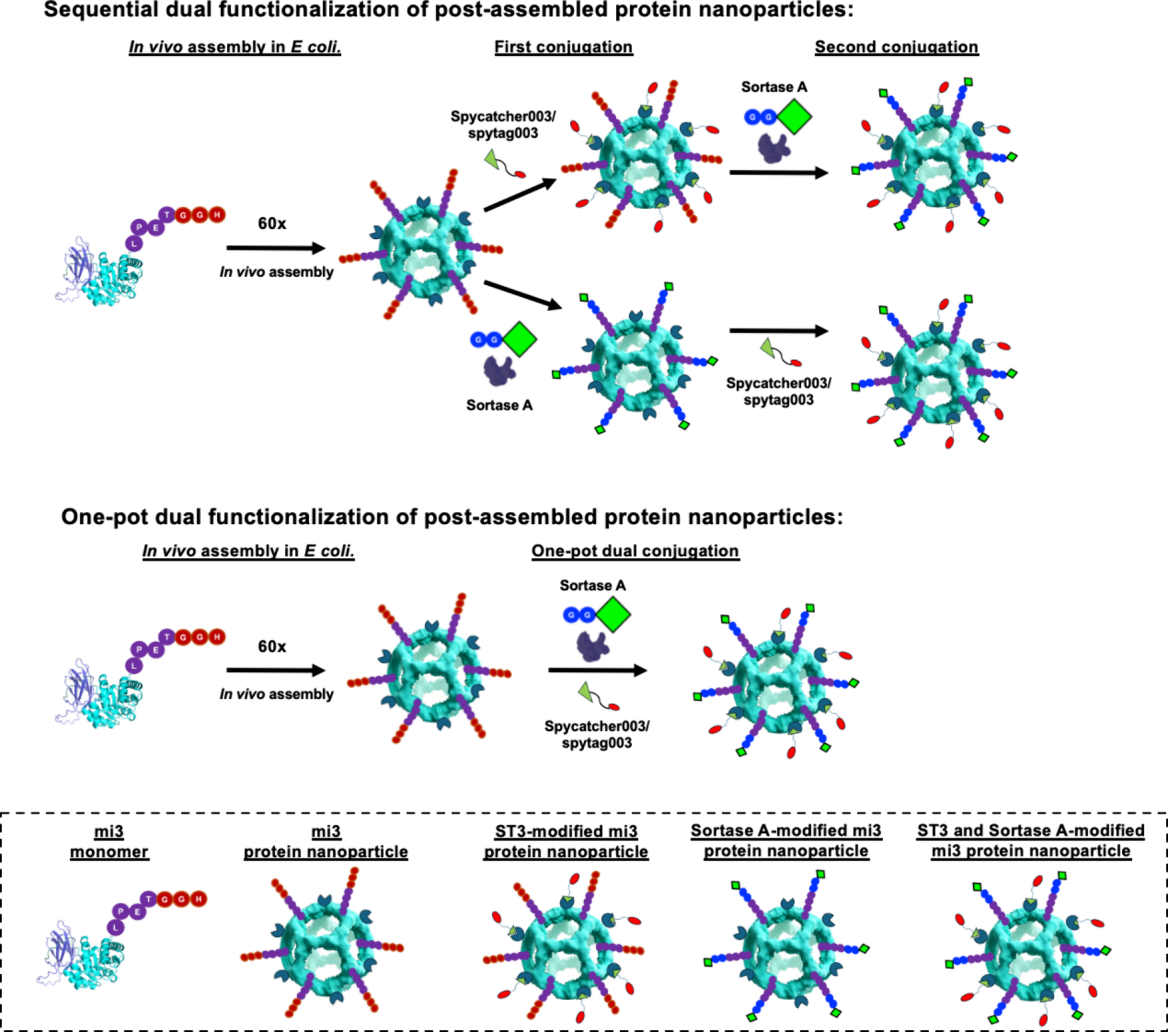
Orthogonal sequential and one-pot dual-functionalization strategies for mi3 self-assembling protein nanoparticles. This schematic delineates two orthogonal bioconjugation strategies, Sortase A-mediated transpeptidation and SpyCatcher003/SpyTag003 (SC3/ST3) isopeptide bond formation, employed for the modular functionalization of engineered mi3 nanoparticles. The foundation of this platform is an mi3 monomer (VNP6-SC3-mi3-LPETGGH), expressed and in vivo assembled in *E. coli*, which comprises an N-terminal VNP6 (vesicle-nucleating peptide) and SpyCatcher003 (SC3) domain, alongside a C-terminal Sortase A recognition motif (LPETGGH). These functional elements are interconnected by flexible Glycine-Serine (Gly-Ser) linkers. The top panel illustrates the sequential dual-functionalization workflow: Preassembled nanoparticles first undergo conjugation with a primary cargo (either via SC3/ST3 with an ST3-modified cargo or Sortase A with an oligoglycine substrate), followed by an essential purification step, and subsequently, a secondary conjugation utilizing the complementary orthogonal system. Conversely, the bottom panel depicts the one-pot dual-functionalization strategy, wherein preassembled nanoparticles are simultaneously incubated with both distinct cargo molecules (ST3-modified and oligoglycine-containing substrates) in the presence of the calcium-independent Sortase A variant. This approach enables concurrent and site-specific ligation without requiring intermediate purification steps.

### Protein Expression and Biophysical Characterization of mi3 Nanoparticles

Initial expression of SC3-mi3-LPETGGH in BL21­(DE3) *E. coli* using standard shaker flask cultivation yielded limited quantities (30–35 mg/L) (Supplementary Data S1). To enhance yields, we incorporated an N-terminal vesicle-nucleating peptide (VNP6),^25^ generating VNP6-SC3-mi3-LPETGGH (Figure 2A). VNP6 was originally engineered to promote intracellular membrane anchoring and secreted expression.^25^ We exploited this property by employing low-temperature expression (16 °C) with IPTG induction to enhance energy-independent intracellular membrane anchoring while suppressing energy-dependent extracellular vesicle secretion. This strategy substantially improved expression yields. Coomassie Blue-stained SDS-PAGE revealed an approximately 3-fold enhancement in protein expression (Figure 2B), yielding ∼100–150 mg/L of purified protein following sequential ammonium sulfate precipitation, anion exchange chromatography, and size-exclusion chromatography (Figure 3).

**Figure 2:**
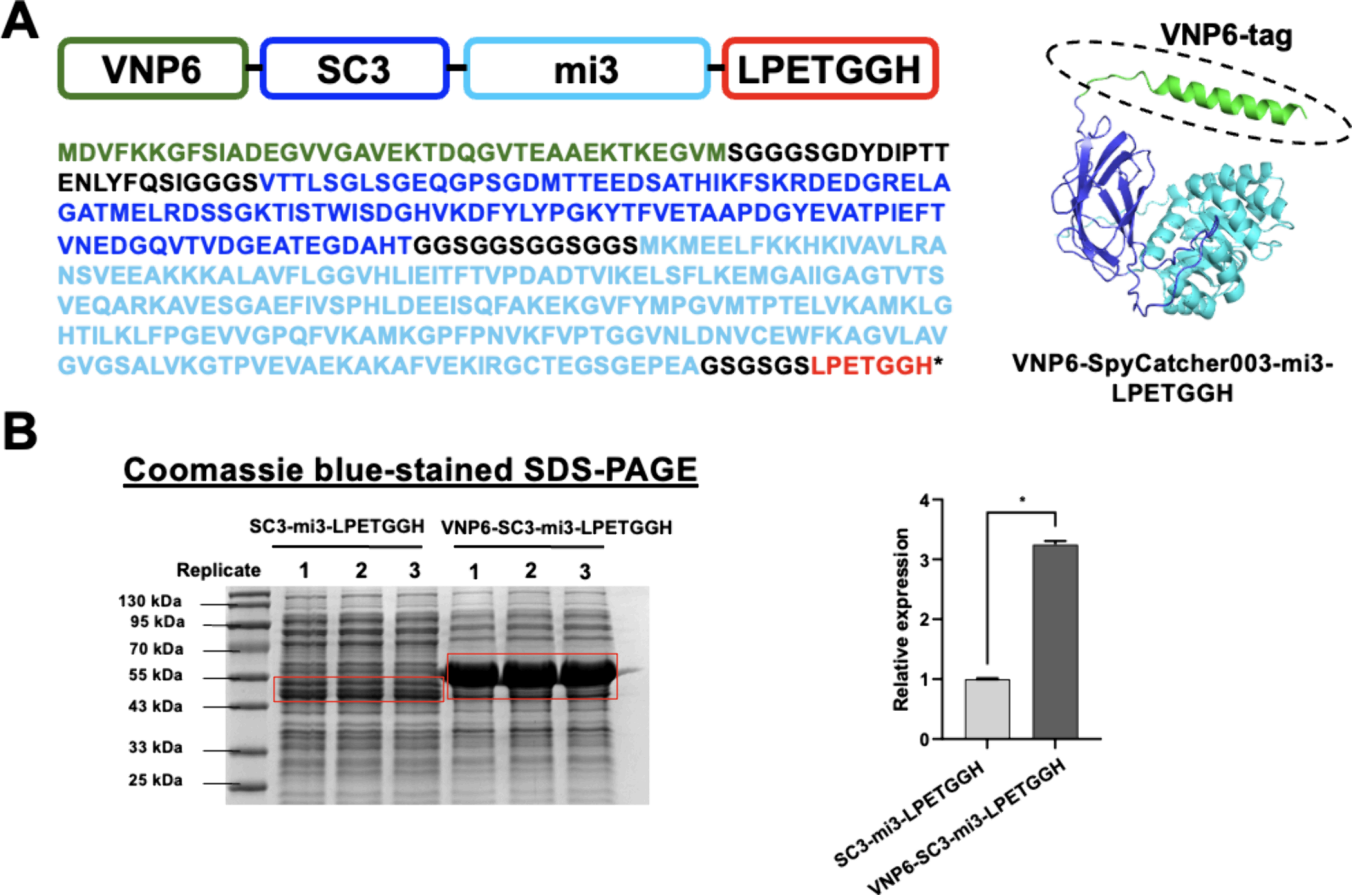
Design and enhanced expression of VNP6-SC3-mi3-LPETGGH nanoparticle subunits. (A) Schematic representation of the primary sequence and AlphaFold2-predicted model of the engineered mi3 nanoparticle subunit. The construct incorporates an N-terminal vesicle-nucleating peptide (VNP6), a SpyCatcher003 (SC3) domain, the mi3 scaffold, and a C-terminal Sortase A recognition motif (LPETGGH). Flexible Gly-Ser linkers are strategically positioned between functional domains to minimize steric hindrance and ensure proper folding and functionality. (B) Coomassie Blue-stained SDS-PAGE analysis comparing soluble protein expression from *E. coli* BL21­(DE3) cultures induced with 0.4 mM IPTG at 16 °C for 16 h expressing either SC3-mi3-LPETGGH or VNP6-SC3-mi3-LPETGGH constructs in triplicate. Densitometry quantification (right panel) demonstrates ∼3-fold enhancement in soluble protein yield with VNP6-incorporated construct (100–150 vs 30–35 mg/L). Data represent mean ± SD (*n* = 3); *p < 0.05 by unpaired Student’s *t* test.

**Figure 3:**
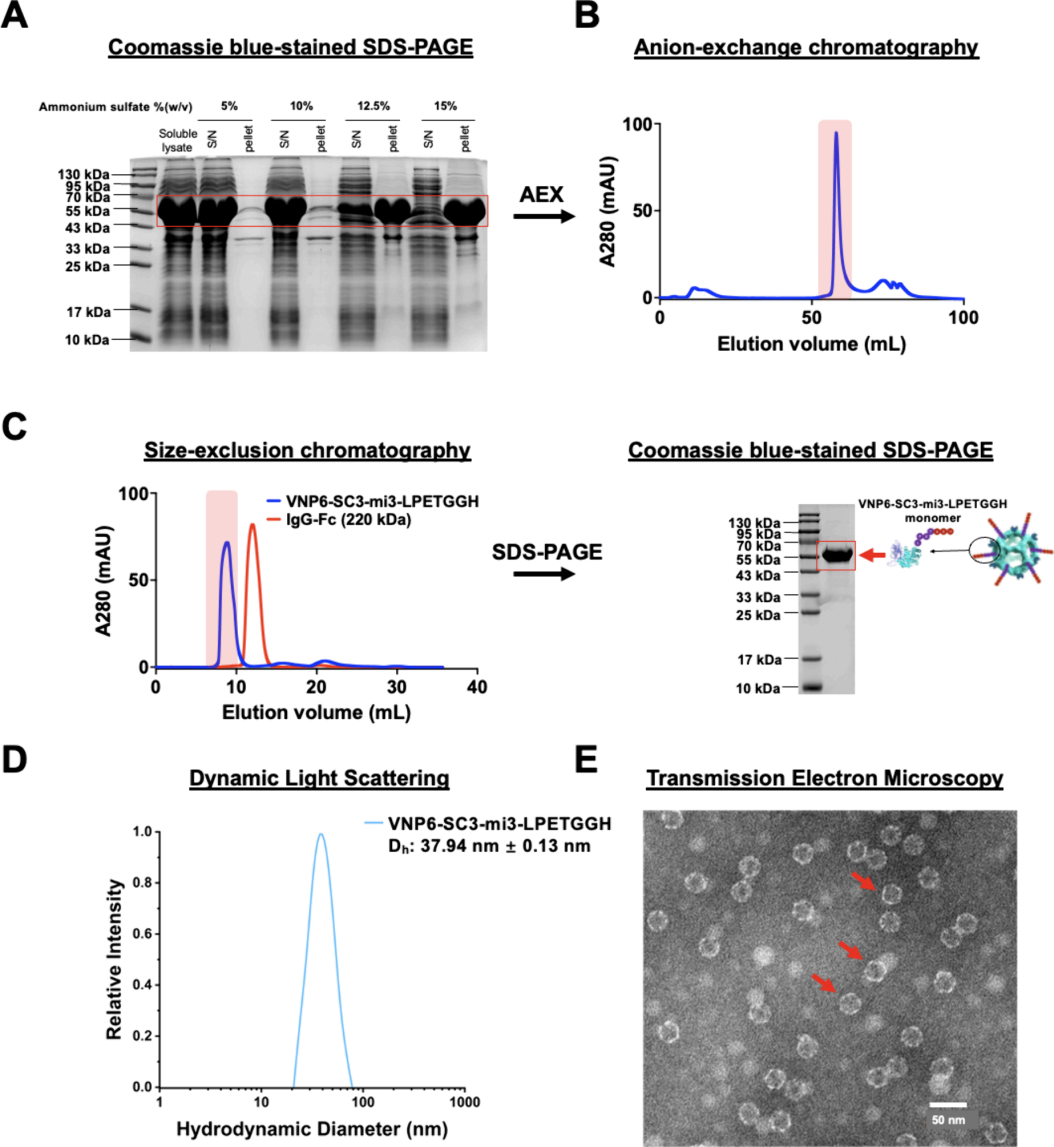
Purification and biophysical characterization of VNP6-SC3-mi3-LPETGGH nanoparticles. (A) Coomassie Blue-stained SDS-PAGE analysis of soluble protein following ammonium sulfate precipitation. (B) FPLC elution profile during anion exchange chromatography, showing a single major peak at ∼0.5 M NaCl. (C) FPLC elution profile following Superdex 200 Increase size-exclusion chromatography with corresponding Coomassie Blue-stained SDS-PAGE analysis of purified fractions; the observed band corresponds to the monomer derived from the protein nanoparticle. (D) Dynamic light scattering (DLS) measurement of purified nanoparticles showing a hydrodynamic radius of 37.9 ± 0.1 nm (*n* = 3). (E) Representative transmission electron microscopy (TEM) image of negatively stained nanoparticles displaying intact cage-like structures. Scale bar, 50 nm.

Purified VNP6-SC3-mi3-LPETGGH retained full assembly competency. Biophysical characterization confirmed nanoparticle integrity. Size-exclusion chromatography (SEC) revealed a homogeneous assembly profile with elution earlier than the IgG-Fc marker (∼220 kDa), consistent with a large supramolecular complex. Dynamic light scattering (DLS) indicated a monodisperse population with an average hydrodynamic radius of 37.94 ± 0.13 nm. Transmission electron microscopy (TEM) showed a well-defined intact cage-like structures (Figure 3). These results confirm successful incorporation of functional tags while maintaining nanoparticle structural integrity.

### Sequential Dual Functionalization Using SC3/ST3 and Sortase A Systems

To establish sequential dual-functionalization capability, we first synthesized a bifunctional fluorescent-biotin peptide probe (GGYSK­(Biotin)­K­(FAM)) via Fmoc-based solid-phase peptide synthesis, followed by TFA cleavage, RP-HPLC purification, and LC-QTOF-MS characterization (Supplementary Data S2).

Initial conjugation experiments employed a functionally active Sortase A pentamutant (eSrt).^23^ However, conjugation attempts proved inefficient as the nanoparticles precipitated under the millimolar calcium ion concentrations required for optimal enzymatic activity (Supplementary Data S3 and S4). Although EDTA supplementation partially mitigated precipitation by chelating excess divalent ions, the fundamental calcium dependency of eSrt remained a significant limitation (Supplementary Data S4). To address this challenge, we expressed SRT7+, a calcium-independent Sortase A variant with a substituted calcium-binding domain.^19^ We further optimized protein expression by incorporating the VNP6 tag, generating VNP6-SRT7+, which substantially enhanced protein yields while preserving enzymatic ligation activity (Supplementary Data S5).

VNP6-SRT7+-mediated conjugation of VNP6-SC3-mi3-LPETGGH with the fluorescent peptide probe (GGYSK­(Biotin)­K­(FAM)) was performed in PBS (pH 7.4). Reactions reached saturation within 30–60 min, yielding VNP6-SC3-mi3-LPETGGYSK­(Biotin)­K­(FAM) nanoparticles, confirmed by fluorescence gel imaging (Figure 4A,B). UV–vis spectroscopy analysis revealed that ∼39.2% of the available C-terminal LPETG sites on the mi3 nanoparticles were successfully modified with the fluorescent peptide. Subsequent SC3/ST3 ligation was performed by incubating the purified, fluorescently labeled nanoparticles with affibody-ST3 constructs (Z_wt_-ST3 or HER2-binding Z_Her2_-ST3) in PBS (pH 7.4) for 60 min (Figure 4C). Successful conjugation was confirmed by green fluorescence gel imaging and Western blot analysis, which revealed characteristic band shifts corresponding to the molecular weights of the conjugated affibody-ST3 fusion proteins (Figure 4D). These results demonstrate sequential dual-functionalization capability of the mi3 platform.

**Figure 4:**
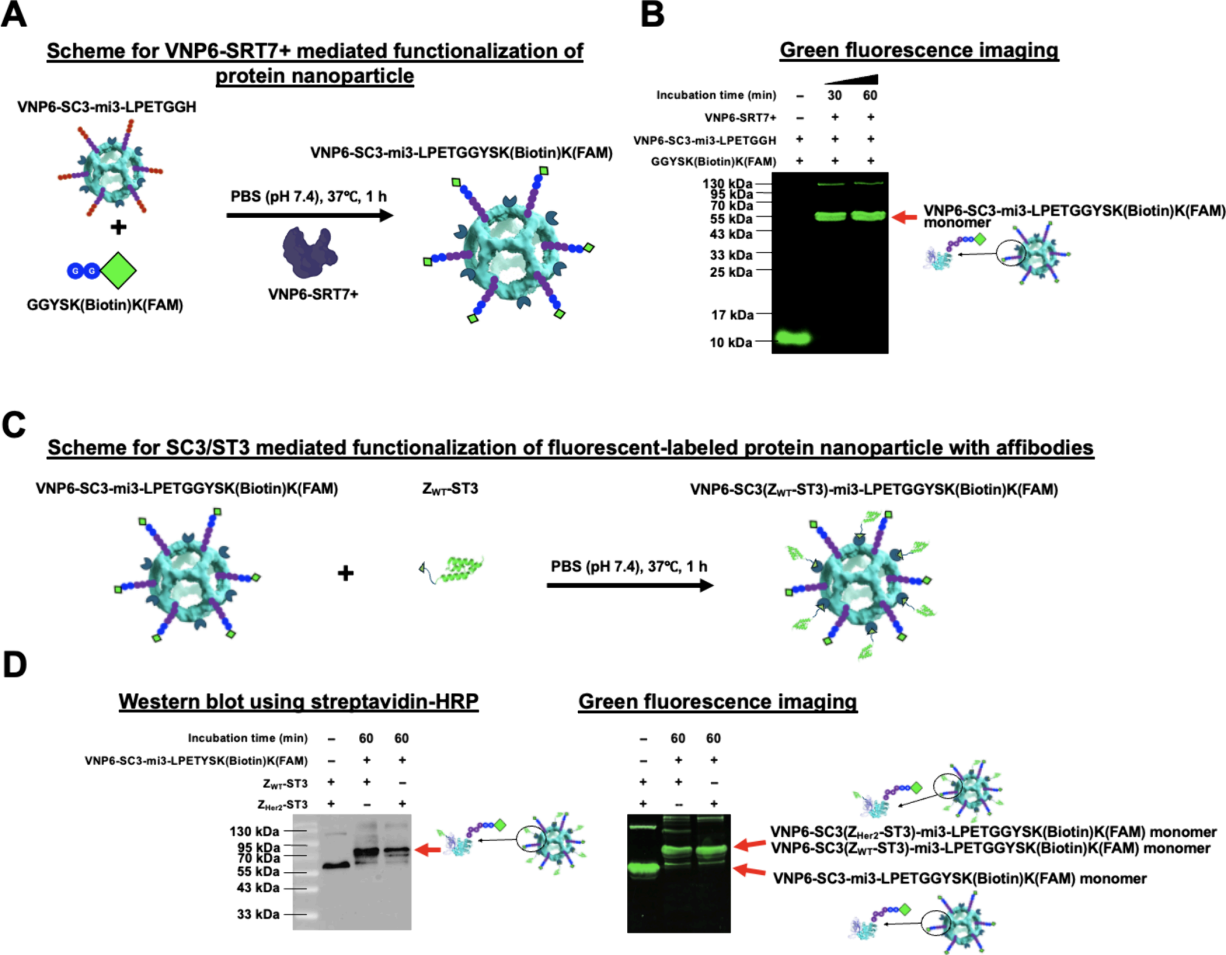
Sequential dual functionalization demonstrates independent compatibility of orthogonal ligation systems. (A) Reaction scheme showing Sortase A-mediated transpeptidation of VNP6-SC3-mi3-LPETGGH nanoparticles with the GGYSK­(Biotin)­K­(FAM) peptide probe. (B) Green fluorescence SDS-PAGE imaging of VNP6-SC3-mi3-LPETGGH nanoparticles following Sortase A-catalyzed conjugation. Green fluorescence signal indicates successful incorporation of the FAM-labeled peptide probe. The observed band corresponds to the monomer derived from the protein nanoparticle. (C) Reaction scheme depicting subsequent SC3/ST3-mediated ligation of fluorescently labeled nanoparticles with wild-type control affibody (Z_wt_-ST3; the observed band corresponds to the monomer derived from the protein nanoparticle. (D) Western blot using streptavidin-HRP and green fluorescence imaging analysis of VNP6-SC3-mi3-LPETGGH nanoparticles following SC3/ST3-mediated functionalization with Z_wt_-ST3. Molecular weight shift (∼20 kDa increase) indicates successful affibody conjugation; the observed band corresponds to the monomer derived from the protein nanoparticle.

### One-Pot Orthogonal Dual Functionalization

Building on the buffer compatibility of both conjugation methods in PBS (pH 7.4), we developed a one-pot protocol for simultaneous dual functionalization. VNP6-SC3-mi3-LPETGGH nanoparticles were incubated with ST3 affibody (Z_wt_-ST3), fluorescent peptide probe (GGYSK­(Biotin)­K­(FAM)), and VNP6-SRT7+ in a single-reaction mixture in PBS (pH 7.4). Both orthogonal conjugation reactions proceeded simultaneously without observable crosstalk (Figure 5A). Reactions reached saturation within 30–60 min, as evidenced by distinct band shifts on SDS-PAGE corresponding to Z_wt_-ST3 conjugation, concurrent with green fluorescence labeling confirming successful peptide probe ligation (Figure 5B,C).

**Figure 5:**
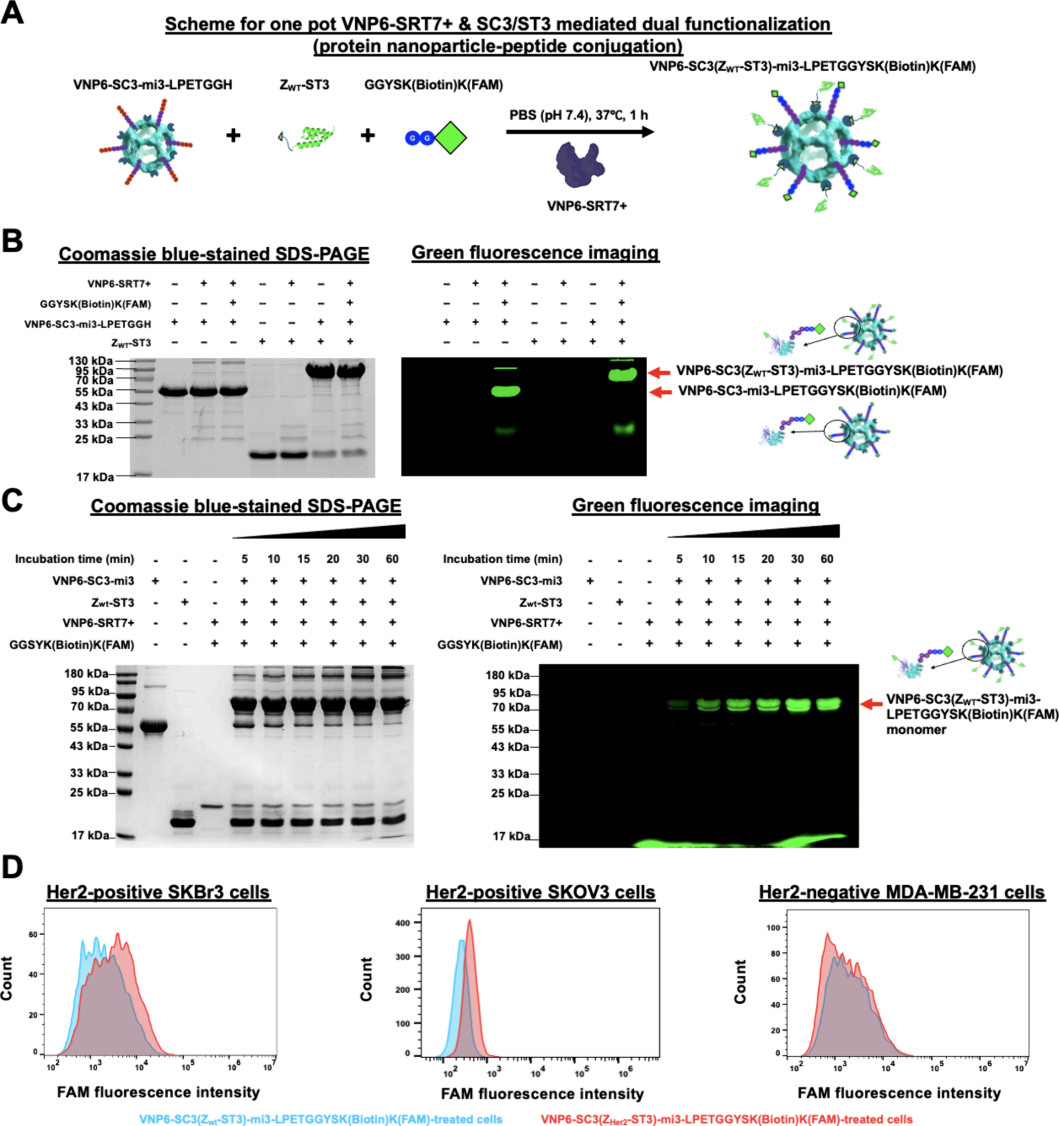
One-pot dual functionalization achieves simultaneous orthogonal ligation and demonstrates HER2-targeted cellular selectivity. (A) Reaction scheme for one-pot dual functionalization of VNP6-SC3-mi3-LPETGGH nanoparticles combining Sortase A-mediated transpeptidation with the GGYSK­(Biotin)­K­(FAM) peptide probe and SC3/ST3-mediated conjugation with Z_wt_-ST3 affibody without intermediate purification. (B) Coomassie Blue-stained SDS-PAGE (left) and green fluorescence imaging (right) of one-pot dual-functionalized nanoparticles following 60 min incubation at 37 °C. Molecular weight shift indicates successful SC3/ST3 conjugation (∼20 kDa increase), while green fluorescence confirms concurrent Sortase A-catalyzed FAM-peptide incorporation. The observed band corresponds to the monomer derived from the protein nanoparticle. (C) Time-course analysis (0, 15, 30, 45, and 60 min) by Coomassie Blue-stained SDS-PAGE and green fluorescence imaging demonstrates reaction progression and saturation within 30–60 min. The observed band corresponds to the monomer derived from the protein nanoparticle. (D) Flow cytometry analysis of cellular uptake in HER2-positive SKBr3, HER2-positive SKOV3, and HER2-negative MDA-MB-231 cells following incubation with 1 μM Z_wt_-ST3 (control) or 1 μM Z_Her2_-ST3 (HER2-targeted) functionalized nanoparticles medium for 60 min at 37 °C. Data were analyzed using FlowJo software.

Affibodies are small three-helix bundle proteins derived from the Z-domain of Staphylococcal protein A, which can be engineered to bind diverse molecular targets with high affinity and specificity.^26–28^ In this study, we employed ZHer2-ST3, which specifically targets the HER2 receptor, and the wild-type Zwt-ST3 serves as a nontargeting control.^29^ To demonstrate functional integrity of the dually functionalized platform, we performed one-pot dual functionalization with either Z_wt‑_ST3 (control) or HER2-binding Z_Her2_-ST3 alongside the fluorescent peptide probe, yielding VNP6-SC3­(Z_wt_-ST3)-mi3-LPETGGYSK­(Biotin)­K­(FAM) or VNP6-SC3­(Z_Her2_-ST3)-mi3-LPETGGYSK­(Biotin)­K­(FAM) nanoparticles (Supplementary Data S6). HER2-positive SKBr3, SKOV3, and HER2-negative MDA-MB-231 cells were treated with these constructs, and cellular uptake was quantified by flow cytometry. HER2-positive SKBr3 and SKOV3 cells treated with Z_Her2_-ST3–functionalized nanoparticles exhibited higher mean fluorescence intensity (MFI) compared to Z_wt_-ST3 controls, whereas HER2-negative MDA-MB-231 cells showed equivalent MFI between the two constructs (Figure 5D). These results confirm that HER2-targeting affinity is retained after dual functionalization and that cellular uptake is HER2-dependent.

### Versatility of One-Pot Dual Functionalization with Protein Cargo

To further demonstrate the versatility and generalizability of the one-pot approach, we employed a larger protein substrate, GGG-SC3-mNeonGreen3A (mNG3A), as a model for diverse cargo types (Figure 6A). Notably, the GGG-SC3-mNG3A cargo itself incorporates an SC3 domain, establishing a branched, multivalent linkage. Following Sortase A ligation of GGG-SC3-mNG3A to the mi3 C-terminus, each functionalized mi3 monomer uniquely presents two SC3 domains: one natively at its N-terminus (derived from the VNP6-SC3-mi3 construct) and a second on the newly appended GGG-SC3-mNG3A at its C-terminus. Consequently, an ST3 affibody (e.g., Z_wt_-ST3) can conjugate to both available SC3 sites on a single mi3 monomer. Coomassie Blue-stained SDS-PAGE analysis revealed that concurrent application of VNP6-SRT7+ and the SC3/ST3 system enables efficient and orthogonal dual functionalization of VNP6-SC3-mi3-LPETGGH nanoparticles. The resulting band patterns corresponded to VNP6-SC3-mi3-LPETGGH monomers conjugated with both Z_wt_-ST3 (via SC3/ST3 ligation) and mNG3A (via Sortase A transpeptidation), though we observed a lower overall functionalization efficiency for the bulkier GGG-SC3-mNG3A cargo (Figure 6B).

**Figure 6:**
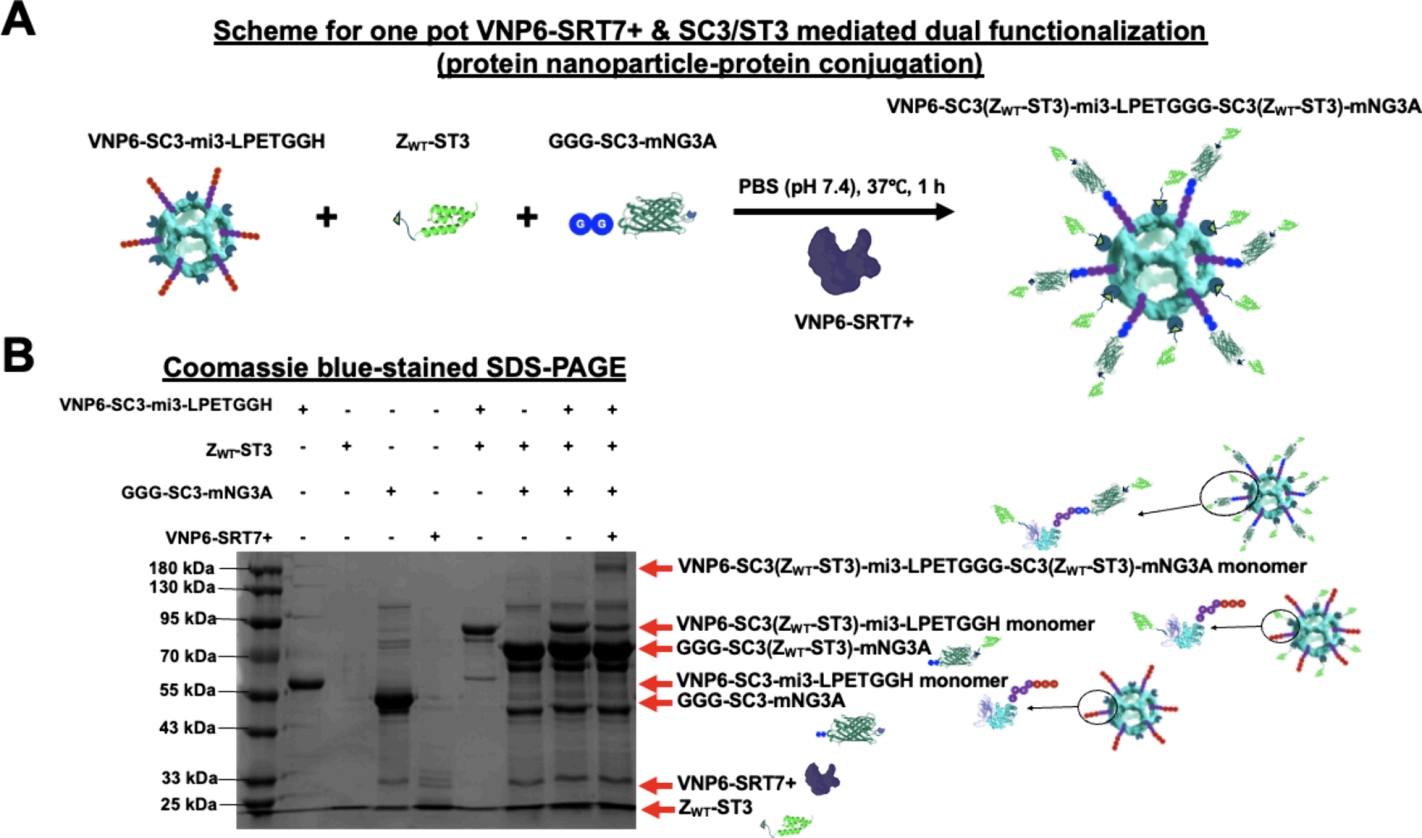
Versatility of one-pot dual functionalization platform with diverse protein cargo. (A) Reaction scheme demonstrating one-pot dual functionalization of VNP6-SC3-mi3-LPETGGH nanoparticles with large protein cargo (GGG-SC3-mNG3A, ∼42 kDa) and Z_wt_-ST3 affibody control simultaneously via Sortase A and SC3/ST3 mechanisms. (B) Coomassie Blue-stained SDS-PAGE analysis showing successful incorporation of both large protein substrate (GGG-SC3-mNG3A, ∼42 kDa) via Sortase A-mediated ligation and affibody (Z_wt_-ST3, ∼20 kDa) via SC3/ST3-mediated conjugation. Molecular weight shift (∼82 kDa increase) indicates successful conjugation of two affibody and one large protein substrate (GGG-SC3-mNG3A) per monomer, with one affibody attaching to the VNP6-SC3-mi3 scaffold and the second to the newly appended GGG-SC3-mNG3A cargo.

## Discussion

We developed an integrated self-assembling protein nanoparticle platform combining Sortase A-mediated transpeptidation and SpyCatcher003/SpyTag003 (SC3/ST3) conjugation on computationally designed mi3 self-assembling protein nanoparticles for orthogonal dual functionalization in one pot. This approach leverages the mechanistic orthogonality between the two systems. Sortase A catalyzes enzymatic transpeptidation while SC3/ST3 spontaneously forms irreversible isopeptide bonds, preventing cross-reactivity and enabling selective modification at spatially separated sites.^13,20,21^ Performing both reactions concurrently in PBS (pH 7.4) without intermediate purification steps enhances the practical feasibility of multivalent nanoparticle conjugation. This integrated approach establishes a robust, scalable, and modular platform that expands the utility and accessible functionalization strategies for mi3 nanoparticles. Such precise, multisite modification in a single step is critical for developing advanced bionanomaterials for diverse applications, including targeted drug delivery, vaccine design, and integrated diagnostics.

The orthogonal combination of SC3/ST3 and Sortase A-based systems presents distinct advantages for simultaneous multicargo functionalization of protein nanoparticles. SC3/ST3 operates through catalyst-free isopeptide bond formation, while Sortase A catalyzes enzyme-mediated transpeptidation. These mechanistically distinct reactions proceed in parallel without crosstalk.^13,21,22^ Additionally, spatial separation of modification sites (N-terminal for SC3/ST3, C-terminal for Sortase A via LPETG motifs) provides orthogonal positioning. Realizing this integrated platform required two key optimizations. First, incorporating the vesicle-nucleating peptide (VNP6) at the N-terminus increased protein yields approximately 3-fold from 30–35 to 100–150 mg/L. Low-temperature expression at 16 °C leverages VNP6’s membrane-anchoring properties to improve solubility while suppressing energy-dependent vesicle secretion.^25^ Importantly, this enhancement preserved nanoparticle assembly integrity, confirmed by transmission electron microscopy showing intact cage-like structures and dynamic light scattering showing a hydrodynamic radius of 37.94 ± 0.13 nm. The successful implementation of VNP6 to increase soluble expression yield thus provides an important bioengineering tool for achieving practical protein yields essential for downstream applications. Second, we adopted SRT7+, a calcium-independent Sortase A variant, rather than calcium-dependent forms such as eSrt or wild-type SrtA. Millimolar calcium concentrations required by traditional Sortase A variants induced mi3 precipitation, severely limiting conjugation efficiency and reaction homogeneity. Although EDTA partially mitigated this issue, the fundamental calcium dependency remained problematic. SRT7+ catalyzes efficient transpeptidation in standard PBS buffers without additives or precipitation, directly enabling one-pot integration with SC3/ST3 reactions.

Our experimental validation confirms the successful implementation of both sequential and one-pot orthogonal ligation strategies. Sequential conjugation was achieved by first performing SRT7+-mediated attachment of the fluorescent peptide GGYSK­(Biotin)­K­(FAM) to the mi3 nanoparticles, followed by purification of the fluorescently labeled nanoparticles, and then subsequent SC3/ST3-mediated affibody attachment. This functionalization was confirmed by SDS-PAGE band shifts and fluorescence imaging. Conversely, the one-pot dual conjugation, where both reactions proceeded simultaneously, yielded identical functionalization results within 30–60 min, demonstrating the orthogonality of these two ligation systems. Notably, larger protein substrates such as GGG-SC3-mNG3A were successfully incorporated, validating compatibility with diverse cargo sizes. Flow cytometry analysis confirmed that dually functionalized nanoparticles retained full HER2-targeting specificity, with higher cellular uptake in HER2-positive SKBr3 and SKOV3 cells compared to HER2-negative MDA-MB-231 cells and Z_wt_-ST3 controls. This preservation of ligand binding affinity validates that the one-pot strategy maintains biological functionality.

While existing mi3 functionalization methods are effective, particularly those using large protein "catcher" systems, our platform presents a versatile alternative with a comparatively smaller functional footprint. The versatility of this platform was demonstrated through the successful conjugation of diverse molecular cargoes, ranging from small peptide probes to larger protein domains. The ability to simultaneously functionalize the nanocage with a tumor-targeting moiety (anti-HER2 affibody) and an imaging agent (fluorophore) underscores the platform’s potential in biomedical applications, particularly in targeted drug delivery, integrated theranostics, vaccine development, and nanozymes. By facilitating the coattachment of therapeutic payloads and targeting ligands on a single structural scaffold, this strategy significantly enhances specificity and the therapeutic index. Beyond clinical uses, this dual-functionalization approach holds promise for enzyme immobilization in nanobioreactors, and programmable nanoassembly. While one-pot systems may yield lower overall conversion efficiencies compared to optimized sequential reactions, they offer distinct practical advantages highly relevant for application development. By consolidating multiple conjugation steps, this method streamlines workflows, reduces hands-on time, and minimizes material losses associated with intermediate purification steps (e.g., ultrafiltration or SEC) in sequential approaches, thereby improving process efficiency. These benefits collectively contribute to a more efficient and cost-effective overall process, even if conversion yield may warrant further optimization for specific applications.

Despite these strengths, limitations remain, particularly regarding potential steric constraints imposed by bulky cargoes, which may affect loading stoichiometry and surface homogeneity, as exemplified by the GGG-SC3-mNG3A results in this study. One factor contributing to the observed efficiencies, particularly for Sortase A-mediated ligation (SML), is its inherent reversibility. Specifically, the ligated product can sometimes regenerate an LPETG-like motif (e.g., LPETGG) that remains susceptible to Sortase A-mediated reverse cleavage, contributing to an equilibrium that limits maximal surface modification. Additionally, complex factors such as surface overcrowding, reduced accessibility of the mi3 C-terminal LPETG-like motif, steric hindrance from multidomain protein cargoes, and potential effects on cargo solubility or stability at high valency can collectively influence ligation yields, particularly with bulky substrates.

The SML efficiency reported herein reflects a strategic compromise made to establish a functional one-pot system for mi3 nanoparticles. Our primary objective was to overcome the calcium-induced aggregation of mi3 by Sortase A. This necessitated the selection of the calcium-independent SRT7+ variant, despite the knowledge that more active calcium-dependent variants could offer higher intrinsic ligation efficiencies. However, these variants are incompatible with mi3 nanoparticles because the calcium concentrations required for their high ligation efficiency induce mi3 precipitation. Therefore, the observed efficiency with SRT7+ directly stems from this strategic decision, prioritizing overall platform feasibility and stability over maximal single-step ligation efficiency in this initial demonstration. While not optimal, this study’s primary aim was to demonstrate the feasibility and orthogonality of integrating these two distinct systems and resolving the mi3 calcium precipitation challenge.

Improving SML efficiency represents a key area for future investigation. This includes engineering more active calcium-independent Sortase A variants, employing strategies to reduce reverse reactions (e.g., leaving group quenching), and systematically optimizing linker lengths and reaction conditions for both the mi3 nanoparticle and cargoes. Beyond this, future efforts should focus on tailoring reaction kinetics for enhanced control and investigating steric hindrances with larger biomolecules Furthermore, this strategy could be scaled up, extended to alternative scaffolds such as ferritins and encapsulins, or adapted for biocompatible live-cell surface functionalization. Finally, the incorporation of additional orthogonal protein ligases with different substrate specificity, such as peptide asparaginyl ligases and connectases, could expand the platform’s capability beyond dual functionalization.^30–35^

In conclusion, our one-pot dual functionalization approach, leveraging orthogonal Sortase A and SC3/ST3 ligation systems, represents an advancement toward creating a robust, ″off-the-shelf″ multifunctional self-assembling protein nanoparticle platform. These technical advancements provide insights into bioconjugation design, extending beyond prior mi3 functionalizations by enabling scalable, biocompatible modifications under mild conditions. This versatile, user-friendly strategy streamlines the assembly of protein nanoparticles with diverse bioactive functionalities, paving the way for accelerated development of sophisticated theranostics, targeted nanomedicines, and beyond.

## Materials and Methods

### Materials

All plasmid constructs for protein expression were commercially synthesized by GenScript (China). Unless otherwise specified, all remaining chemicals and solvents were purchased from Aladdin (China) or Macklin (China).

### Recombinant Protein Expression

The pET29b expression plasmids encoding mi3 variants (SC3-mi3-LPETGGH, VNP6-SC3-mi3-LPETGGH) and His-tagged recombinant proteins (Sortase A variants including eSrt, SRT7+, and VNP6-SRT7+; affibodies Z_wt_-ST3 and Z_Her2_-ST3; and protein substrates GGG-SC3-mNG3A and IL-1Ra-LPETGGH) were transformed into *E. coli* BL21­(DE3) cells. Transformed cells were plated on LB agar containing 50 μg/mL kanamycin and incubated overnight at 37 °C. Single colonies were inoculated into 4 mL of LB medium supplemented with 50 μg/mL kanamycin and incubated for 4 h at 37 °C with shaking at 200 rpm. Precultures were subsequently diluted into 1 L of fresh LB medium (with 50 μg/mL kanamycin) and grown at 37 °C (200 rpm) until an OD_600_ of 0.6 was reached. Protein expression was induced by the addition of 0.4 mM IPTG for 16 h at 16 °C. Bacterial cultures were harvested by centrifugation at 6000 × *g* for 20 min at 4 °C.

### Purification of mi3 Nanoparticles

Bacterial cell pellets from cultures expressing SC3-mi3-LPETGGH or VNP6-SC3-mi3-LPETGGH were resuspended in lysis buffer (50 mM HEPES, 0.1 M NaCl, 1 mM EDTA, 5% glycerol, 5 mM β-mercaptoethanol, 0.1% Triton X-100, pH 7.5) supplemented with 1 mM PMSF. Cells were lysed by sonication (30% amplitude, 3 s on/7 s off cycle for 10 min), and the lysate was clarified by centrifugation (12,000 × *g*, 20 min, 4 °C). The supernatant was filtered through a 0.22 μm syringe filter (Biosharp, China). Ammonium sulfate was added to the filtrate with constant mixing for 1 h at 4 °C, followed by centrifugation at 12,000 × *g* for 20 min at 4 °C. The resulting pellet was resuspended in PBS (pH 7.4) and filtered through a 0.22 μm syringe filter.

Strong anion exchange chromatography was performed using Q-NUPharose (NUPTEC, China) on a FPLC system. Elution was accomplished using a linear gradient of 1 M NaCl in PBS (pH 7.4) over eight column volumes (CV) at 1 mL/min. The eluent was dialyzed and concentrated using Amicon Ultra-15 centrifugal filters (10 kDa MWCO, Millipore, USA). Size-exclusion chromatography (SEC) was performed on a Superdex 200 Increase 10/300 GL column (Cytiva, Sweden) with PBS as the mobile phase. Fractions were analyzed by SDS-PAGE with Coomassie blue staining. Protein concentration was determined using the Bradford assay. Purified proteins were stored at −20 °C in storage buffer (50 mM Tris, 150 mM NaCl, 10% (v/v) glycerol, pH 8.0).

### Purification of His-Tagged Recombinant Proteins

Bacterial cell pellets containing His-tagged proteins were resuspended in lysis buffer and disrupted by ultrasonication. Lysates were clarified by centrifugation at 12,000 × *g* for 20 min at 4 °C and filtered through a 0.22 μm syringe filter (Biosharp, China). The supernatant was incubated with 1 mL of pre-equilibrated nickel-nitrilotriacetic acid (Ni-NTA) immobilized metal affinity chromatography (IMAC) resin (GenScript, China) for 8 h at 4 °C with rotation. The resin was washed with 10 CV of wash buffer (10 mM Na_2_HPO_4_, 137 mM NaCl, 5 mM β-mercaptoethanol, 30 mM imidazole, pH 7.4). Proteins were eluted with 10 CV of elution buffer (10 mM Na_2_HPO_4_, 137 mM NaCl, 5 mM β-mercaptoethanol, 250 mM imidazole, pH 7.4). Eluted fractions were analyzed by SDS-PAGE and pooled. For the GGG-SC3-mNG3A construct, the protein was designed with an N-terminal methionine–glycine–glycine–glycine start sequence to ensure that the endogenous *E. coli* methionine aminopeptidase would remove the initial methionine, thereby exposing the N-terminal GGG motif required for subsequent Sortase A ligation. Buffer exchange into phosphate buffer (10 mM Na_2_HPO_4_, 137 mM NaCl, pH 7.4) was performed using Amicon Ultra-15 centrifugal filters (10 kDa MWCO, Millipore, USA). Protein concentrations were determined by the Bradford assay, and samples were stored at −20 °C in storage buffer (50 mM Tris, 150 mM NaCl, 10% (v/v) glycerol, pH 8.0).

### Dynamic Light Scattering (DLS)

To determine the hydrodynamic radius, protein samples were centrifuged at 16,000 × *g* for 60 min at 4 °C to remove any aggregates. DLS measurements were performed on a Zetasizer Ultra instrument (Malvern Panalytical, UK) at 20 °C, recording 10 scans of 10 s each.

### Negative Staining and Transmission Electron Microscopy (TEM) Imaging

Five microliters of the sample was deposited on the carbon-coated grid and incubated for 1 min. Excess sample was blotted with Whatman paper. The grid was stained with 2% (w/v) uranyl acetate, and excess stain was removed by blotting. Imaging was performed on a Hitachi 7400 transmission electron microscope operating at 80 kV.

### Fmoc-Based Solid-Phase Peptide Synthesis

The GGYSK­(Biotin)­K­(FAM)-NH_2_ peptide was synthesized by fluoren-9-ylmethoxycarbonyl (Fmoc)-based solid-phase peptide synthesis (SPPS) on Fmoc-Rink-amide resin with a mixture of Fmoc-amino acids (4.0 equiv), *N*,*N*-diisopropylethylamine (DIPEA; 6.0 equiv), (benzotria-zol-1-yloxy)­tripyrrolidinophosphonium hexafluorophosphate (PyBOP; 4.0 equiv) in dimethylformamide (DMF) for 2 h at room temperature as previously described.^36,37^ The synthesized peptide was cleaved by treatment with cleavage solution (92.5% TFA, 2.5% H_2_O, 2.5% 1,2-ethanedithiol, 2.5% triisopropylsilane) at room temperature for 2 h, followed by precipitation with ice-cold *tert*-butyl methyl ether. The GGYSK­(Biotin)­K­(FAM)-NH_2_ peptide was then purified by preparative RP-HPLC on a C18 column (10 mm × 250 mm; 10 μm particle size, Phenomenex, USA) using a linear gradient of 5–95% acetonitrile in 0.1% TFA. Identity was confirmed by LC-QTOF-MS.

### Sequential Dual Functionalization of VNP6-SC3-mi3-LPETGGH Nanoparticles

#### Sortase A-Mediated Conjugation

A reaction mixture containing 1 μM VNP6-SC3-mi3-LPETGGH nanoparticles, 2 μM GGYSK­(Biotin)­K­(FAM)-NH_2_, and 1 μM VNP6-SRT7+ was incubated in PBS (pH 7.4) at 37 °C for 60 min. The reaction was analyzed by SDS-PAGE using green fluorescence imaging and Coomassie blue staining. To isolate the product, the VNP6-SC3-mi3-LPETGGYSK­(Biotin)­K­(FAM) product was purified by size-exclusion chromatography on an ENrich SEC 75 column (Bio-Rad, USA) at 0.4 mL/min. Target fractions were pooled and concentrated using Amicon Ultra-15 filters (100 kDa MWCO, Millipore, USA).

#### SpyCatcher003/SpyTag003-Mediated Conjugation

For the subsequent SpyCatcher003/SpyTag003 (SC3/ST3) ligation step, 1 μM of the purified VNP6-SC3-mi3-LPETGGYSK­(Biotin)­K­(FAM) nanoparticles were incubated with 4 μM Z_wt_-ST3 or 10 μM Z_Her2_-ST3 in PBS (pH 7.4) at 37 °C for 60 min. The mixture was resolved by SDS-PAGE and analyzed by green fluorescence imaging and Western blot. For Western blotting, proteins were transferred to a 0.45 μm PVDF membrane (Millipore, USA) at 300 mA for 100 min on ice. The membrane was blocked with 5% bovine serum albumin (BSA) in TBST (Tris-buffered saline +0.1% Tween 20) and incubated overnight at 4 °C with Streptavidin-HRP (1 μg/mL in 5% BSA-TBST). The membrane was washed five times with TBST (10 min each) and visualized using a chemiluminescence substrate (MedChemExpress, China) on a ChemiDoc imaging system (Bio-Rad, USA).

### One-Pot Dual Functionalization of VNP6-SC3-mi3-LPETGGH Nanoparticles

A reaction mixture containing 1 μM VNP6-SC3-mi3-LPETGGH nanoparticles, 2 μM affibody fusion (Z_wt_-ST3 or Z_Her2_-ST3), 2 μM GGYSK­(Biotin)­K­(FAM)-NH_2_, and 1 μM VNP6-SRT7+ was incubated in PBS (pH 7.4) at 37 °C for 60 min. Both orthogonal conjugation reactions (Sortase A-mediated transpeptidation and SC3/ST3 isopeptide bond formation) proceeded simultaneously in a single vessel without intermediate purification steps. To test the system with larger protein cargoes, the peptide probe was replaced with 10 μM GGG-SC3-mNG3A in an identical reaction mixture. Reaction mixtures were analyzed by SDS-PAGE with Coomassie blue staining and green fluorescence imaging.

### Degree of Labeling

The conjugation efficiency of the sortagged sites on the mi3 nanoparticles was quantified by determining the Degree of Labeling via UV–vis spectrophotometry. The molar concentration of conjugated FAM was calculated from its absorbance at 495 nm according to the Beer–Lambert law. To determine the corrected protein monomer concentration, the measured absorbance at 280 nm was corrected for FAM spectral overlap using a correction factor of 0.3 and subsequently divided by the extinction coefficient of the monomer. Finally, the Degree of Labeling was calculated as the percentage ratio of the molar concentration of conjugated FAM to that of the protein monomer.

### Cell Culture

HER2-positive SKBr3, SKOV3, and HER2-negative MDA-MB-231 cells were cultured in DMEM/F12 supplemented with 10% FBS and 100 U/mL penicillin/streptomycin in a humidified incubator with 5% CO_2_ at 37 °C. Cells between passages 5 and 20 were used for all experiments. For uptake experiments, cells were seeded at a density of 1 × 10^5^ cells/mL per well for 24 h prior to nanoparticle treatment.

### Cellular Uptake Analysis by Flow Cytometry

HER2-positive SKBr3, SKOV3, and HER2-negative MDA-MB-231 cells were incubated with 1 μM VNP6-SC3­(Z_wt_-ST3)-mi3-LPETGGYSK­(Biotin)­K­(FAM) or VNP6-SC3­(Z_Her2_-ST3)-mi3-LPETGGYSK­(Biotin)­K­(FAM) in DMEM/F12 medium for 60 min at 37 °C. Following incubation, cells were trypsinized and resuspended in DMEM/F12 complete medium. A total of 10,000 cells per sample were analyzed using a BD LSR FACSCelesta flow cytometer.

### Statistical Analysis

All quantitative data are expressed as mean ± standard deviation (SD) unless otherwise noted. Statistical analyses were performed using GraphPad Prism 9 (GraphPad Software, San Diego, California, USA). Comparative analyses between two groups were conducted using unpaired Student’s *t* tests (two-tailed, *n* = 3). Statistical significance was defined as *p* < 0.05.

## Supplementary Material



## Notes

Protein sequences; purification of SC3-mi3-LPETGGH protein; synthesis and characterization of GGYSK­(Biotin)­K­(FAM)-NH2 peptide probe; Sortase A-mediated functionalization of SC3-mi3-LPETGGH nanoparticles with GGYSK­(Biotin)­K­(FAM) peptide probe; calcium-induced precipitation of VNP6-SC3-mi3-LPETGGH nanoparticles; recombinant expression and activity test of VNP6-SRT7+; and one-pot dual functionalization of VNP6-SC3-mi3-LPETGGH nanoparticles with affibodies and GGYSK­(Biotin)­K­(FAM) peptide probe (PDF)
