# Supplementary material for: One-Pot Orthogonal Dual Functionalization of mi3 Self-Assembling Protein Nanoparticles via Sortase A and SpyCatcher/SpyTag Ligation
Source: Bioconjug Chem. 2026 Jun 25;37(7):1409–20. doi: 10.1021/acs.bioconjchem.6c00173 (PMC13377597; doi:10.1021/acs.bioconjchem.6c00173)
Supplement: Supplementary file 1 [file bc6c00173_si_001.pdf]

# One-Pot Orthogonal Dual Functionalization of mi3 Self-Assembling Protein Nanoparticles via Sortase A and SpyCatcher/SpyTag Ligation

Liqiang Wei[1,2], Hongfei Wang[1], Chunyue Du[2], Antony Kam[2,*], Shining Loo[1,*]

[1] Wisdom Lake Academy of Pharmacy, Xi'an Jiaotong-Liverpool University, Wuzhong No.111, Renai Road, Suzhou, Jiangsu, 215123, People's Republic of China

[2] Department of Biosciences and bioinformatics, School of Science, Xi'an Jiaotong-Liverpool University, Wuzhong No.111, Renai Road, Suzhou, Jiangsu, 215123, People's Republic of China

* Correspondence:

Dr. Antony Kam, Department of Biosciences and bioinformatics, School of Science, Xi'an Jiaotong-Liverpool University, Wuzhong No. 111, Renai Road, Suzhou, Jiangsu, 215123, People's Republic of China. Email: Antony.Kam@xjtlu.edu.cn

Dr. Shining Loo, Wisdom Lake Academy of Pharmacy, Xi'an Jiaotong-Liverpool University, Wuzhong No. 111, Renai Road, Suzhou, Jiangsu, 215123, People's Republic of China. Email: Shining.Loo@xjtlu.edu.cn

**Protein sequences**

***SC3-mi3-LPETGGH***

MGLGSSVTTLSGLSGEQGPSGDMTTEEDSATHIKFSKRDEDGRELAGATMELRDSSG
KTISTWISDGHVKDFYLYPGKYTFVETAAPDGYEVATPIEFTVNEDGQVTVDGEATEGD
AHTGGSGGSGGSGGSMKMEELFKKHKIVAVLRANSVEEAKKKALAVFLGGVHLIEITF
TVPDADTVIKELSFLKEMGAIIGAGTVTSVEQARKAVESGAEFIVSPHLDEEISQFAKEK
GVFYMPGVMTPTELVKAMKLGHTILKLFPGEVVGPQFVKAMKGPFPNVKFVPTGGVN
LDNVCEWFKAGVLAVGVGSALVKGTPVEVAEKAKAFVEKIRGCTEGSGEPEAGSGSG
SLPETGGH*

***VNP6-SC3-mi3-LPETGGH***

MDVFKKGFSIADEGVVGAVEKTDQGVTEAAEKTKEGVMSGGGSGDYDIPTTENLYFQ
SIGGGSVTTLSGLSGEQGPSGDMTTEEDSATHIKFSKRDEDGRELAGATMELRDSSG
KTISTWISDGHVKDFYLYPGKYTFVETAAPDGYEVATPIEFTVNEDGQVTVDGEATEGD
AHTGGSGGSGGSGGSMKMEELFKKHKIVAVLRANSVEEAKKKALAVFLGGVHLIEITF
TVPDADTVIKELSFLKEMGAIIGAGTVTSVEQARKAVESGAEFIVSPHLDEEISQFAKEK
GVFYMPGVMTPTELVKAMKLGHTILKLFPGEVVGPQFVKAMKGPFPNVKFVPTGGVN
LDNVCEWFKAGVLAVGVGSALVKGTPVEVAEKAKAFVEKIRGCTEGSGEPEAGSGSG
SLPETGGH*

***eSrtA***

MQAKPQIPKDKSKVAGYIEIPDADIKEPVYPGPATREQLNRGVSFAEENESLDDQNISIA
GHTFIDRPNYQFTNLKAAKKGSMVYFKVGNETRKYKMTSIRNVKPTAVEVLDEQKGKD
KQLTLITCDDYNEETGVWETRKIFVATEVKLEHHHHHH*

***SRT7+***

MDYDIPTTENLYFQSIGGGSQAKPQIPKDKSKVAGYIEIPDADIKEPVYPGPATREQLNR
GVSFAKENQSLDDQNISIAGHTFIGRPNYQFTNLKAAKKGSMVYFKVGNETRKYKMTSI
RNVKPTAVEVLDEQKGKDKQLTLITCDDLNRETGVWETRKIFVATEVKLEHHHHHH*

***VNP6-SRT7+***

MDVFKKGFSIADEGVVGAVEKTDQGVTEAAEKTKEGVMSGGGSGDYDIPTTENLYFQ
SIGGGSQAKPQIPKDKSKVAGYIEIPDADIKEPVYPGPATREQLNRGVSFAKENQSLDD
QNISIAGHTFIGRPNYQFTNLKAAKKGSMVYFKVGNETRKYKMTSIRNVKPTAVEVLDE
QKGKDKQLTLITCDDLNRETGVWETRKIFVATEVKLEHHHHHH*

***IL-1Ra-LPETGGH***

MDVFKKGFSIADEGVVGAVEKTDQGVTEAAEKTKEGVMSGGGSGHHHHHHDYDIPTT
ENLYFQSIGGGSRPSGRKSSKMQAFRIWDVNQKTFYLRNNQLVAGYLQGPNVNLEEK
IDVVPIEPHALFLGIHGGKMCLSCVKSGDETRLQLEAVNITDLSENRKQDKRFAFIRSDS
GPTTSFESAACPGWFLCTAMEADQPVSLTNMPDEGVMVTKFYFQEDELPETGGH*

***GGG-SC3-mNG3A***

MGGGSGHHHHHHDYDIPTTENLYFQSIGGGSVTTLSGLSGEQGPSGDMTTEEDSATH
IKFSKRDEDGRELAGATMELRDSSGKTISTWISDGHVKDFYLYPGKYTFVETAAPDGY
EVATPIEFTVNEDGQVTVDGEATEGDAHTGSSGSVSKGEEDNMASLPATHELHIFGSI
NGVDFDMVGQGTGNPNDGYEELNLKSTKGDLQFSPWILVPHIGYGFHQYLPYPDGMS
PFQAAMVDGSGYQVHRTMQFEDGASLTVNYRYTYEGSHIKGEAQVMGTGFPADGPV
MTNTLTAADLCVSKMTYPNDKTIISTFKWSYTTVNGKRYRSTARTTYTFAKPMAAKYLK
NQPMYVLRKTELKHSMTELNFKEWQKAFTDMM*

***$Z_{Her2}$-ST3***

MDVFKKGFSIADEGVVGAVEKTDQGVTEAAEKTKEGVMSGGGSGHHHHHHDYDIPTT
ENLYFQGGGSGGGVDNKFNKEMRNAYWEIALLPNLNNQQKRAFIRSLYDDPSQSANL
LAEAKKLNDAQAPKGSPANLKALEAQKQKEQRQAAEELANAKKLKEQLEKGSRGVPHI
VMVDAYKRYK

***Z**$_{wt}$**-ST3***

MDVFKKGFSIADEGVVGAVEKTDQGVTEAAEKTKEGVMSGGGSGHHHHHHDYDIPTT
ENLYFQGGGSGGGSIVDNKFNKEQQNAFYEILHLPNLNEEQRNAFIQSLKDDPSQSAN
LLAEAKKLNDAQAPKGSPANLKALEAQKQKEQRQAAEELANAKKLKEQLEKGSRGVP
HIVMVDAYKRYK

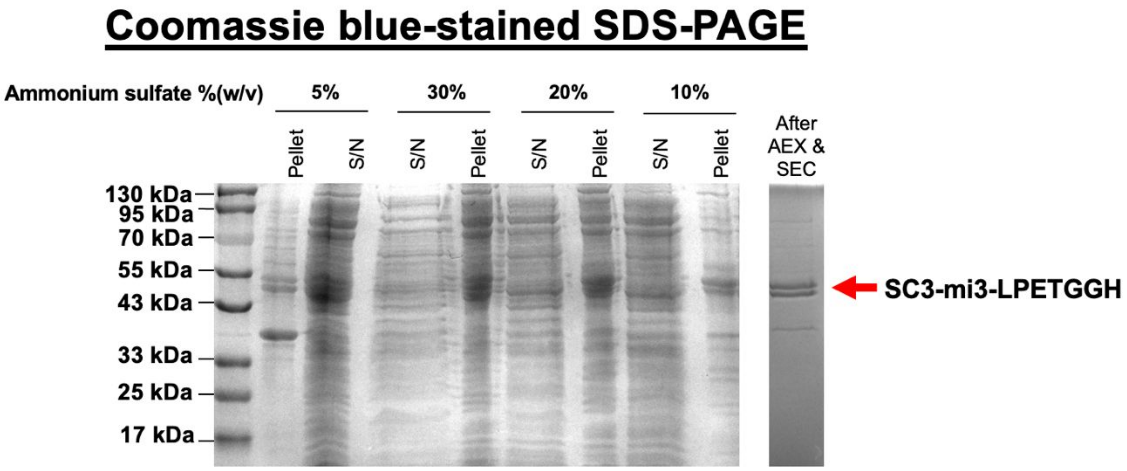


**Supplementary Data S1 Purification of SC3-mi3-LPETGGH protein**. Coomassie Blue-stained SDS-PAGE analysis showing protein at sequential purification stages of ammonium sulfate precipitation and the purified product following anion exchange chromatography and Superdex 200 Increase size-exclusion chromatography.

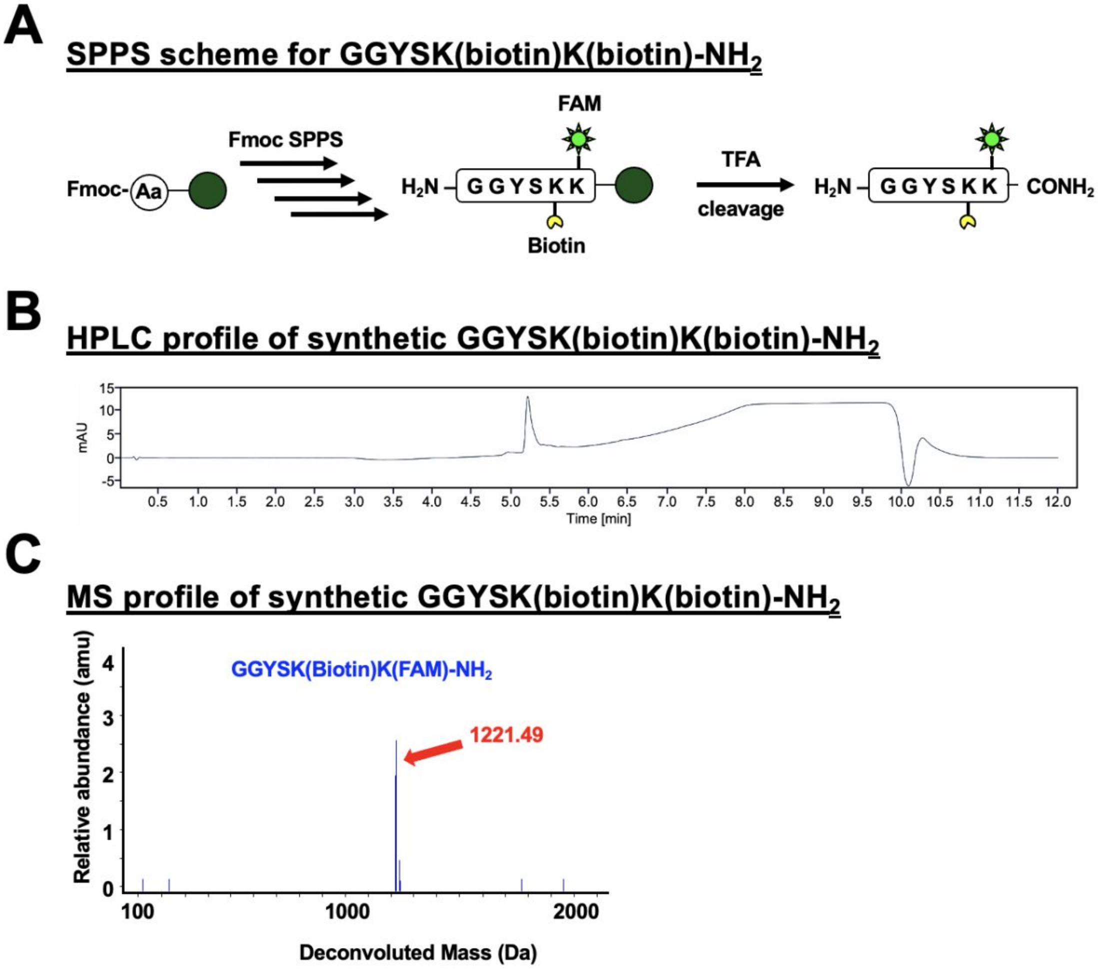


**Supplementary Data S2 Synthesis and characterization of GGYSK(Biotin)K(FAM)-$NH_2$ peptide probe. (A)** Solid-phase peptide synthesis scheme for GGYSK(Biotin)K(FAM)-$NH_2$ peptide probe. **(B)** HPLC profile of purified GGYSK(Biotin)K(FAM)-$NH_2$ peptide probe. **(C)** Mass spectrometry profile of GGYSK(Biotin)K(FAM)-$NH_2$ peptide probe.

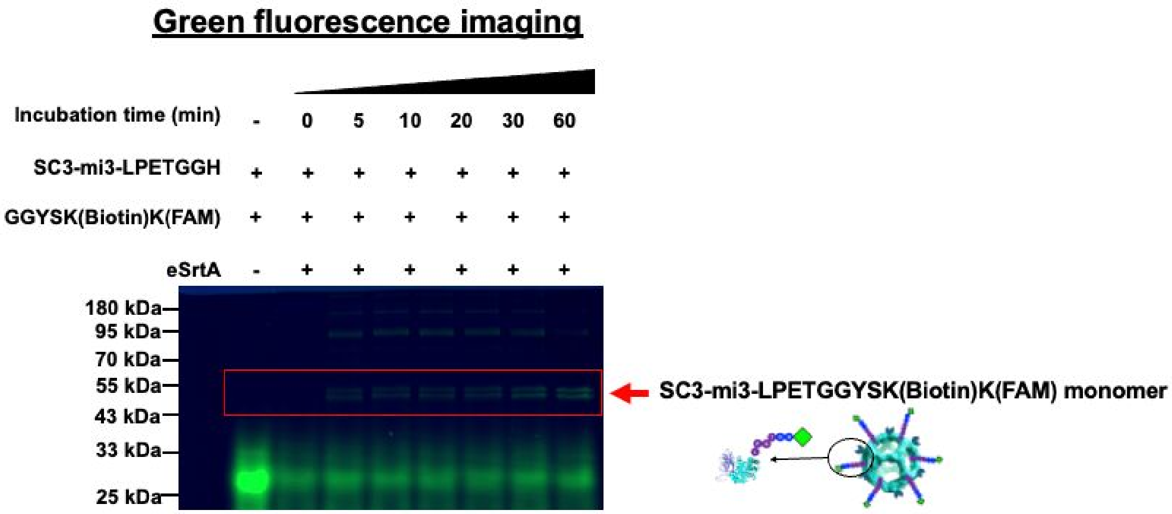


**Supplementary Data S3 Sortase A-mediated functionalization of SC3-mi3-LPETGGH nanoparticles with GGYSK(Biotin)K(FAM) peptide probe.** Green fluorescence SDS-PAGE imaging SC3-mi3-LPETGGH nanoparticles following eSrtA-mediated functionalization with the GGYSK(biotin)K(FAM) peptide probe. Presence of green fluorescence confirms FAM-peptide incorporation.

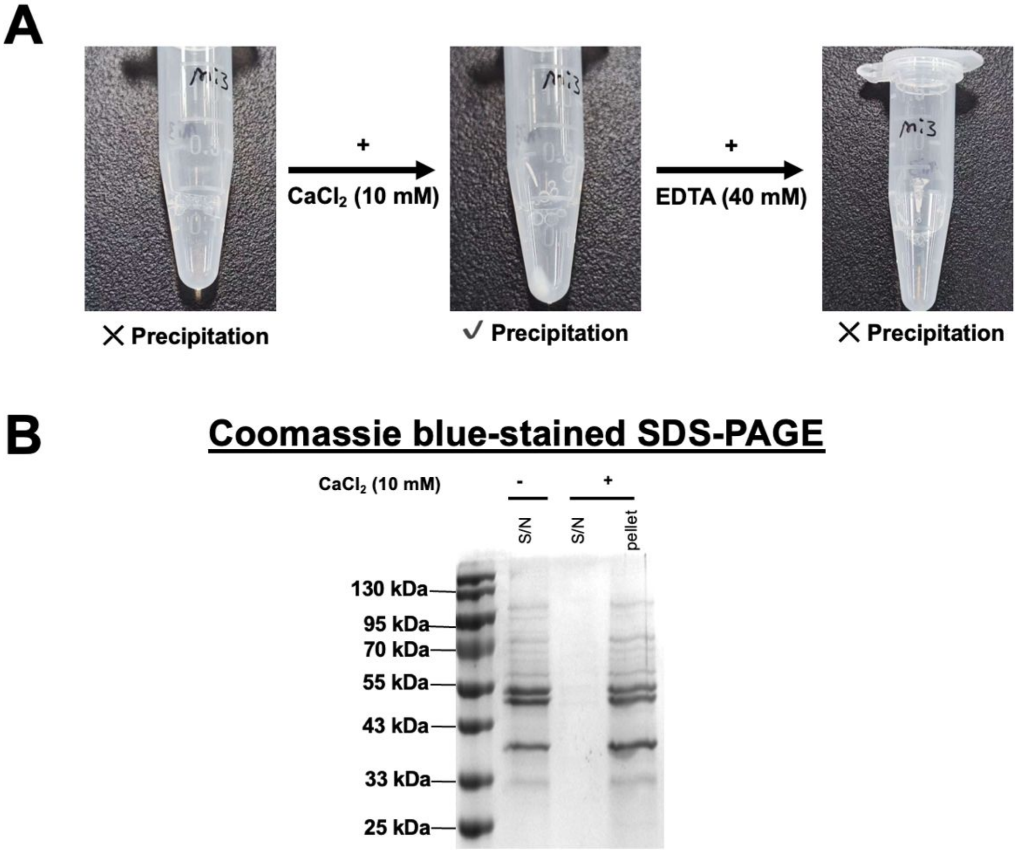


**Supplementary Data S4 Calcium-induced precipitation of VNP6-SC3-mi3-LPETGGH nanoparticles. (A)** Photographs demonstrating $CaCl_2$-mediated VNP6-SC3-mi3-LPETGGH precipitation and its reversibility using EDTA. **(B)** Coomassie Blue-stained SDS-PAGE analysis of VNP6-SC3-mi3-LPETGGH nanoparticles with and without $CaCl_2$ incubation.

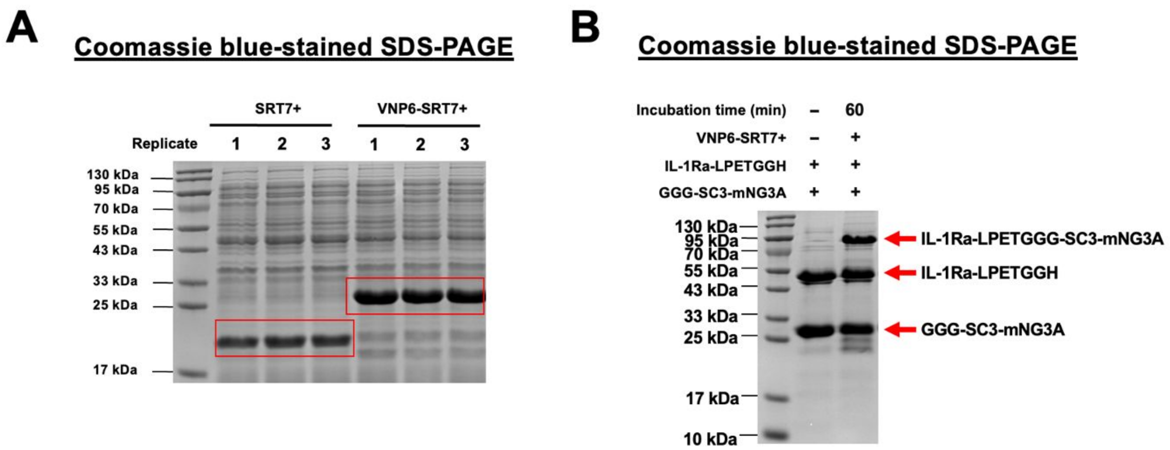


**Supplementary Data S5 Recombinant expression and activity test of VNP6-SRT7+.** **(A)** Coomassie Blue-stained SDS-PAGE analysis of soluble bacterial lysates following IPTG induction of *E. coli* BL21(DE3) transformed with plasmid constructs encoding either VNP6-SRT7+ or SRT7+. **(B)** Coomassie Blue-stained SDS-PAGE analysis of VNP6-SRT7+-mediated conjugation of IL-1Ra-LPETGGH with GGG-SC3-mNG3A protein. Molecular weight increase, as observed by band shift, confirms successful VNP6-SRT7+-mediated conjugation.

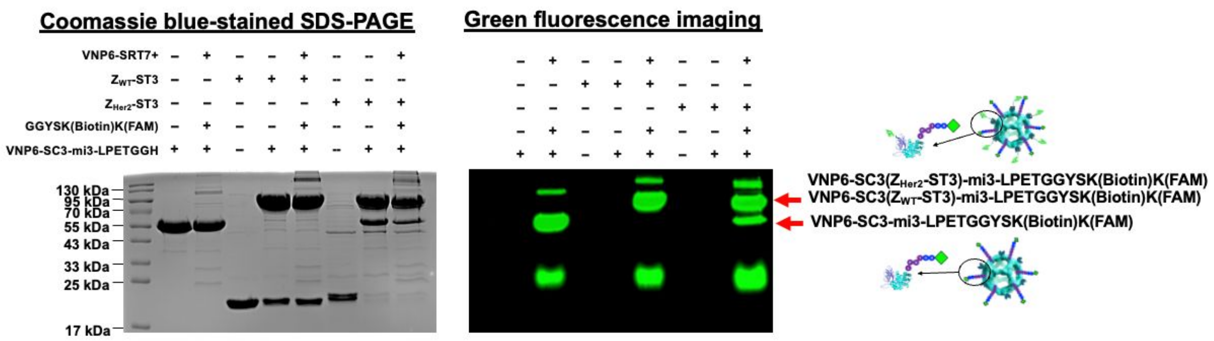


**Supplementary Data S6 One-pot dual functionalization of VNP6-SC3-mi3-LPETGGH nanoparticles with affibodies and GGYSK(Biotin)K(FAM) peptide probe.** Coomassie Blue-stained SDS-PAGE and green fluorescence imaging showing one-pot dual functionalization with GGYSK(Biotin)K(FAM) peptide probe and either $Z_{wt}$-ST3 (wild-type control) or $Z_{Her2}$-ST3 (HER2-targeting variant). Molecular weight increase, as observed by band shift, indicates successful SC3/ST3-mediated affibody ligation. Green fluorescence confirms concurrent VNP6-SRT7+-catalyzed FAM-peptide incorporation in both samples.
